# Supplementary material for: A Likelihood Approach for Real-Time Calibration of Stochastic Compartmental Epidemic Models
Source: PLoS Comput Biol. 2017 Jan 17;13(1):e1005257. doi: 10.1371/journal.pcbi.1005257 (PMC5240920; doi:10.1371/journal.pcbi.1005257)
Supplement: S1 File — (TAR.GZ) [file pcbi.1005257.s014.tar.gz › HSPH_Online-SI-Revision/Additions/IPoi10/n100-extreme-SEITR/n100-extreme-SEITR_BENCH_table-8.pdf]

1	R0	{1.38654, 9.85565}	{2.38536, 17.2321}	{{2.35866, 16.9062}, {2.46533, 18.6082}}	{72.0372, 74.8453}	inf	369.	673.763	{78., 1585.}	90.1819	0.475486	newly	522.	362.627	{37., 819.}	62.2151	0.546702	Reff	1.36736	2.37416	{2.29854, 2.56616}	73.6307	ar	92379.	84881.6	{86983., 90753.}	8.11587
2	R0	{2.36629, 12.2029}	{1.44746, 17.6177}	{{1.30812, 12.6353}, {1.67375, 19.7105}}	{38.8298, 47.8187}	inf	10.	2.5	{0., 6.}	82.1429	1.00706	newly	20.	1.03125	{0., 3.}	94.8438	1.68404	Reff	2.3656	1.44593	{1.22389, 1.66636}	38.877	ar	99022.	26483.3	{1., 67778.}	73.2551
3	R0	{2.32253, 12.836}	{1.44884, 11.8527}	{{1.0158, 8.09734}, {2.21449, 18.33}}	{39.4481, 30.7228}	inf	18.	1.43	{0., 4.}	94.7037	1.87011	newly	26.	1.05	{0., 3.}	95.9615	1.94424	Reff	2.32139	1.44879	{1.01577, 2.21436}	39.4274	ar	98997.	15327.5	{1., 73696.}	84.5172
4	R0	{1.79075, 11.2058}	{1.37684, 13.9202}	{{1.0158, 8.09734}, {1.85141, 19.5412}}	{26.2195, 41.0138}	inf	68.	4.28333	{0., 8.}	95.5842	1.84678	newly	88.	2.8	{0., 8.}	96.8182	2.08883	Reff	1.78676	1.37886	{1.01574, 1.85139}	26.4545	ar	96920.	24572.	{2., 71918.}	74.6471
5	R0	{2.26952, 9.62371}	{1.42337, 11.4263}	{{1.0158, 8.09734}, {2.20466, 17.2398}}	{38.8607, 38.2978}	inf	2.	1.11	{0., 3.}	79.4	1.04167	newly	5.	0.68	{0., 2.}	86.4	1.26695	Reff	2.26934	1.42334	{1.01578, 2.20464}	38.858	ar	98881.	14469.1	{1., 73838.}	85.3672
6	R0	{1.57191, 6.89004}	{1.57198, 15.4856}	{{1.11461, 10.2835}, {2.22641, 19.7105}}	{19.7946, 124.754}	inf	11.	3.	{0., 7.}	78.0303	0.945788	newly	22.	1.79221	{0., 4.}	91.8536	1.50437	Reff	1.57147	1.58098	{1.18977, 2.22625}	18.8574	ar	95046.	31893.5	{1., 79406.}	66.4441
7	R0	{1.99226, 17.2339}	{2.22641, 14.8348}	{{2.22641, 14.8348}, {2.22641, 14.8348}}	{11.753, 13.9208}	inf	713.	867.	{49., 1979.}	70.723	0.567472	newly	648.	539.943	{31., 1236.}	70.9382	0.590555	Reff	1.94486	2.191	{2.14067, 2.2245}	12.6557	ar	97992.	83276.3	{84546., 85196.}	15.0173
8	R0	{2.63369, 15.1879}	{1.45278, 17.3097}	{{1.22395, 12.6353}, {1.75668, 19.7105}}	{44.8386, 22.387}	inf	35.	2.66102	{0., 5.}	94.8827	1.50787	newly	41.	1.47458	{0., 3.}	96.4035	1.75593	Reff	2.6309	1.47372	{1.30805, 1.75657}	43.9842	ar	99451.	30552.2	{1., 68042.}	69.2791
9	R0	{2.53271, 9.90552}	{1.42337, 11.4263}	{{1.0158, 8.09734}, {2.20466, 17.2398}}	{44.0796, 37.2365}	inf	2.	1.19	{0., 3.}	71.5	0.659791	newly	8.	0.78	{0., 2.}	90.25	1.37989	Reff	2.53258	1.42334	{1.01578, 2.20462}	44.0781	ar	99355.	14253.4	{1., 74936.}	85.6541
10	R0	{2.48791, 18.9003}	{1.91767, 15.3966}	{{1.30812, 12.378}, {2.64373, 19.5412}}	{25.5995, 19.6896}	inf	205.	83.2653	{2., 193.}	75.8121	1.00838	newly	173.	50.8367	{1., 120.}	73.2335	1.00599	Reff	2.47216	1.948	{1.23952, 2.41306}	23.1072	ar	99291.	65076.6	{28., 88198.}	34.4588
11	R0	{2.84414, 18.4765}	{1.32172, 17.091}	{{1.18979, 13.6259}, {1.36512, 19.5412}}	{53.5284, 10.8682}	inf	68.	7.07317	{0., 20.}	93.1328	1.64731	newly	76.	3.36585	{0., 8.}	95.5712	1.81733	Reff	2.83774	1.34629	{1.2233, 1.48679}	52.5579	ar	99654.	21887.1	{3., 47792.}	78.0369
12	R0	{2.29369, 14.622}	{1.39974, 17.9813}	{{1.22395, 15.4344}, {1.66638, 19.7105}}	{38.9742, 26.4306}	inf	100.	7.59091	{1., 19.}	94.6164	1.5493	newly	114.	3.43182	{0., 9.}	96.9896	1.95008	Reff	2.28683	1.39595	{1.22627, 1.66618}	38.9571	ar	98982.	31284.6	{6., 67926.}	68.3937
13	R0	{2.73413, 16.7529}	{1.38646, 16.4564}	{{1.10133, 8.51468}, {1.66638, 19.7105}}	{49.2908, 18.4149}	inf	62.	6.80769	{0., 18.}	91.2722	1.40009	newly	67.	3.63462	{0., 10.}	94.5752	1.72785	Reff	2.72932	1.39321	{1.10675, 1.75626}	48.9538	ar	99543.	30359.4	{4., 71521.}	69.5012
14	R0	{1.79554, 16.5943}	{2.45965, 19.0916}	{{2.41419, 18.743}, {2.52006, 19.5955}}	{36.9869, 15.049}	inf	652.	2022.54	{244., 4284.}	157.156	0.461297	newly	541.	940.524	{125., 2059.}	125.382	0.444235	Reff	1.75512	2.37127	{2.28894, 2.50091}	35.1059	ar	96821.	88654.4	{87934., 89612.}	8.43469
15	R0	{1.91429, 13.7771}	{1.94617, 14.0718}	{{1.94617, 14.0718}, {1.94617, 14.0718}}	{1.66537, 2.13906}	inf	266.	423.021	{53., 858.}	75.1044	0.392242	newly	278.	270.25	{38., 509.}	66.8615	0.395145	Reff	1.89691	1.92908	{1.91075, 1.94366}	1.7882	ar	97541.	78160.7	{77800., 78691.}	19.8689
16	R0	{1.42094, 10.5871}	{2.79994, 18.006}	{{2.46246, 15.904}, {2.86501, 18.4249}}	{97.0486, 70.0745}	inf	260.	821.565	{93., 2013.}	155.42	0.44509	newly	379.	454.984	{51., 1074.}	83.356	0.422759	Reff	1.40736	2.75635	{2.31907, 2.85785}	95.8533	ar	93044.	92123.7	{86518., 93190.}	1.08107
17	R0	{1.45515, 6.16466}	{1.42337, 11.4263}	{{1.0158, 8.09734}, {2.20466, 17.2398}}	{26.9201, 88.8019}	inf	5.	1.18	{0., 3.}	76.	0.878633	newly	11.	0.97	{0., 3.}	91.5455	1.5731	Reff	1.45502	1.42334	{1.01578, 2.20455}	26.92	ar	93254.	12203.9	{1., 67998.}	86.9133
18	R0	{2.85669, 13.2416}	{1.57218, 15.7074}	{{1.10133, 9.64612}, {2.12978, 19.7105}}	{44.9679, 28.6938}	inf	3.	2.7027	{0., 6.}	77.1499	0.851758	newly	8.	1.56757	{0., 4.}	80.7432	1.0822	Reff	2.85618	1.61669	{1.18974, 2.22614}	43.4011	ar	99644.	35605.6	{1., 78897.}	64.2671
19	R0	{2.56417, 17.9558}	{1.26189, 15.2678}	{{1.18979, 13.6259}, {1.34361, 18.7523}}	{50.7877, 18.2139}	inf	91.	5.91892	{1., 17.}	96.1813	1.69306	newly	91.	3.40541	{0., 10.}	96.2578	1.77297	Reff	2.55604	1.26828	{1.18929, 1.34358}	50.381	ar	99389.	24129.1	{6., 46560.}	75.7226
20	R0	{2.27517, 11.1864}	{1.53848, 14.9394}	{{1.0158, 8.09734}, {2.1968, 19.7105}}	{33.0457, 46.5089}	inf	8.	1.86765	{0., 4.}	88.3272	1.19707	newly	17.	1.08824	{0., 2.}	93.5986	1.67769	Reff	2.27446	1.5897	{1.10363, 2.19678}	31.03	ar	98894.	23898.	{1., 78155.}	75.8347
21	R0	{2.35735, 10.4735}	{1.45608, 11.8927}	{{1.0158, 8.09734}, {2.2194, 18.7523}}	{39.3633, 36.4416}	inf	5.	1.15	{0., 3.}	78.6	0.996507	newly	5.	0.85	{0., 2.}	87.	1.22891	Reff	2.35707	1.45606	{1.01578, 2.21938}	39.3585	ar	99036.	15596.5	{1., 72255.}	84.2517
22	R0	{1.79551, 11.9179}	{2.54857, 14.6829}	{{2.48103, 13.7658}, {2.86501, 18.4249}}	{41.9415, 23.2002}	inf	143.	262.967	{23., 558.}	83.0792	0.472929	newly	205.	185.217	{17., 415.}	71.7642	0.511271	Reff	1.78616	2.53265	{2.31201, 2.85547}	41.7933	ar	97033.	86632.6	{85046., 93088.}	10.7184
23	R0	{1.23545, 6.31687}	{2.74389, 16.9229}	{{2.48103, 13.7658}, {2.86501, 18.4249}}	{122.096, 167.9}	inf	66.	26.8438	{4., 58.}	79.0396	0.894671	newly	213.	17.3594	{2., 35.}	91.8501	1.3647	Reff	1.23161	2.75292	{2.56696, 2.86472}	123.523	ar	88739.	90495.6	{90016., 93153.}	5.46927
24	R0	{2.24089, 10.1789}	{1.57085, 15.9113}	{{1.15964, 10.7075}, {1.97176, 19.7105}}	{30.7538, 59.1416}	inf	4.	2.14865	{0., 5.}	65.991	0.713306	newly	6.	1.14865	{0., 3.}	80.8559	1.0682	Reff	2.24062	1.53832	{1.18975, 1.97172}	31.4788	ar	98868.	27407.7	{1., 67875.}	72.2785
25	R0	{1.57298, 10.2607}	{1.57111, 17.2757}	{{1.34361, 13.4368}, {2.1968, 19.5412}}	{17.6318, 68.5101}	inf	107.	18.74	{1., 50.}	86.8028	1.2502	newly	146.	10.	{0., 30.}	93.1507	1.58425	Reff	1.56721	1.64095	{1.34255, 2.19654}	19.2854	ar	94957.	48644.3	{7., 84522.}	48.7723
26	R0	{1.2154, 8.2576}	{2.86701, 18.44}	{{2.86501, 18.4249}, {2.86501, 18.4249}}	{135.891, 123.309}	inf	374.	441.652	{30., 874.}	71.9242	0.454678	newly	559.	241.879	{17., 475.}	70.5806	0.682293	Reff	1.19834	2.84744	{2.82622, 2.86429}	137.616	ar	88421.	93041.	{92841., 93207.}	5.22495
27	R0	{2.22841, 18.7983}	{2.4257, 15.6891}	{{2.32014, 14.7828}, {2.57305, 16.884}}	{8.89097, 17.1743}	inf	579.	844.091	{139., 2070.}	78.9001	0.430262	newly	487.	510.545	{106., 1282.}	79.2346	0.41155	Reff	2.1875	2.40229	{2.25736, 2.54706}	9.91042	ar	98756.	88220.6	{86367., 90110.}	10.6681
28	R0	{1.27778, 5.36767}	{1.59498, 12.657}	{{1.0158, 8.09734}, {2.45321, 19.391}}	{36.2992, 138.489}	inf	15.	2.27	{0., 5.}	87.5	1.41272	newly	37.	1.83	{0., 5.}	95.0541	1.93128	Reff	1.27717	1.5949	{1.01578, 2.45306}	36.3283	ar	89843.	26991.6	{1., 88521.}	70.2447
29	R0	{2.38042, 15.8901}	{1.43337, 16.7611}	{{1.18158, 11.0915}, {1.67375, 19.7105}}	{39.7852, 20.455}	inf	75.	8.17647	{0., 23.}	93.5107	1.7503	newly	93.	4.58824	{0., 13.}	95.0664	1.92745	Reff	2.37421	1.49424	{1.30778, 1.67331}	37.0635	ar	99112.	34724.8	{2., 67987.}	64.9641
30	R0	{2.53188, 12.7607}	{1.42337, 11.4263}	{{1.0158, 8.09734}, {2.20466, 17.2398}}	{44.0632, 32.2701}	inf	1.	1.09	{0., 3.}	78.2	0.946615	newly	3.	0.93	{0., 3.}	77.	0.901196	Reff	2.5317	1.42334	{1.01577, 2.20464}	44.0606	ar	99317.	15252.1	{1., 74940.}	84.643
31	R0	{1.24482, 10.5471}	{2.77354, 19.4633}	{{2.42074, 15.7308}, {2.81635, 19.898}}	{122.807, 84.5365}	inf	904.	3172.36	{749., 6302.}	165.732	0.398992	newly	1087.	1506.31	{448., 2935.}	70.656	0.273361	Reff	1.19689	2.6344	{2.38807, 2.77129}	120.103	ar	89097.	92175.2	{92094., 92739.}	3.63766
32	R0	{2.30446, 17.9607}	{2.73016, 16.9769}	{{2.48103, 13.7658}, {2.86501, 18.4249}}	{18.7529, 9.04642}	inf	288.	104.19	{11., 218.}	76.1972	0.821643	newly	254.	66.5	{8., 161.}	75.7195	0.808077	Reff	2.28427	2.72895	{2.4687, 2.86427}	19.5993	ar	99028.	91613.2	{88735., 93186.}	7.48755
33	R0	{2.31579, 11.8236}	{1.53745, 12.4416}	{{1.0158, 8.09734}, {2.22832, 19.391}}	{35.7286, 32.5047}	inf	22.	3.36	{0., 11.}	89.7143	1.79034	newly	33.	2.58	{0., 7.}	93.697	1.97856	Reff	2.31442	1.53733	{1.01577, 2.22761}	35.7024	ar	99012.	19552.8	{1., 84283.}	80.2521
34	R0	{1.6678, 10.6744}	{2.71473, 17.5734}	{{2.32014, 14.7828}, {2.86501, 18.4249}}	{62.7733, 64.6315}	inf	174.	106.	{8., 271.}	67.5836	0.697659	newly	267.	61.3134	{4., 153.}	77.7852	0.953014	Reff	1.65861	2.71902	{2.31654, 2.86435}	63.9338	ar	95809.	90020.7	{86534., 93151.}	6.04147
35	R0	{1.65476, 7.07559}	{1.45608, 11.8927}	{{1.0158, 8.09734}, {2.2194, 18.7523}}	{26.8495, 71.9444}	inf	1.	1.47	{0., 3.}	82.375	1.08636	newly	10.	1.	{0., 3.}	90.4	1.46632	Reff	1.65458	1.45605	{1.01578, 2.21938}	26.8476	ar	95746.	16283.3	{1., 78280.}	82.9932
36	R0	{2.71807, 18.5959}	{1.37361, 18.1144}	{{1.22395, 15.6333}, {1.56495, 19.5412}}	{49.4639, 5.87727}	inf	74.	4.57895	{0., 10.}	94.8551	1.57743	newly	61.	1.94737	{0., 4.}	96.8076	1.88472	Reff	2.71258	1.3854	{1.18946, 1.56482}	48.9268	ar	99540.	25377.2	{3., 48891.}	74.5056
37	R0	{2.6202, 12.7552}	{1.42825, 17.4315}	{{1.13239, 13.8982}, {1.75668, 19.7105}}	{45.4906, 38.9044}	inf	2.	4.67241	{0., 11.}	66.2562	0.552622	newly	7.	2.36207	{0., 6.}	73.1527	0.834629	Reff	2.61991	1.50383	{1.30248, 1.8513}	42.6	ar	99426.	34780.3	{2., 73454.}	65.0189
38	R0	{1.47672, 9.81651}	{2.60307, 16.974}	{{2.32014, 14.7828}, {2.86656, 18.9813}}	{76.2735, 72.9125}	inf	196.	284.439	{53., 653.}	64.1038	0.393194	newly	329.	162.123	{26., 361.}	63.1259	0.537928	Reff	1.46491	2.61891	{2.3052, 2.86212}	78.7765	ar	93815.	90349.9	{86423., 93152.}	3.69357
39	R0	{1.90004, 12.3313}	{1.51572, 15.9726}	{{1.20956, 12.378}, {1.91377, 19.7105}}	{22.0827, 33.1736}	inf	121.	11.9474	{1., 36.}	93.0539	1.50816	newly	167.	5.97368	{0., 17.}	96.4229	1.9278	Reff	1.89271	1.57108	{1.23936, 2.22432}	20.979	ar	97413.	45630.	{4., 78388.}	53.1582
40	R0	{2.64218, 18.5237}	{2.32741, 17.1681}	{{1.34361, 12.6353}, {2.86501, 18.7523}}	{21.0432, 8.32259}	inf	208.	98.386	{1., 255.}	73.766	1.02765	newly	187.	56.4561	{0., 134.}	75.6638	1.09802	Reff	2.62693	2.43986	{1.34356, 2.86404}	18.4595	ar	99474.	74994.9	{9., 93183.}	24.6085
41	R0	{1.6945, 7.17996}	{1.45608, 11.8927}	{{1.0158, 8.09734}, {2.2194, 18.7523}}	{27.331, 69.5903}	inf	3.	1.42	{0., 4.}	75.2	0.934742	newly	9.	1.03	{0., 4.}	88.5556	1.3686	Reff	1.69433	1.45605	{1.01577, 2.21936}	27.3291	ar	96332.	13912.8	{1., 71108.}	85.5574
42	R0	{1.84559, 18.0074}	{2.77291, 19.53}	{{2.76795, 19.6041}, {2.81635, 19.898}}	{50.2453, 9.97261}	inf	1046.	1391.58	{97., 3925.}	79.3874	0.535103	newly	870.	670.638	{44., 1856.}	74.8026	0.567192	Reff	1.78061	2.71317	{2.58299, 2.80452}	52.3733	ar	97358.	92199.1	{92070., 92702.}	5.2989
43	R0	{2.24965, 17.0361}	{2.79606, 18.0169}	{{2.32014, 14.7828}, {2.86501, 18.4249}}	{24.6478, 9.20175}	inf	172.	178.155	{9., 434.}	64.0282	0.550778	newly	161.	99.1379	{4., 269.}	64.1893	0.623097	Reff	2.23721	2.79593	{2.47974, 2.86424}	24.9912	ar	98770.	89207.5	{86531., 93183.}	9.68162
44	R0	{2.54324, 15.7967}	{1.34217, 17.1415}	{{1.04117, 8.84894}, {1.66638, 19.5412}}	{47.2259, 21.6771}	inf	68.	5.89362	{1., 12.}	94.9627	1.54592	newly	107.	3.04255	{0., 10.}	97.1565	1.939	Reff	2.53714	1.32689	{1.04113, 1.56476}	47.7012	ar	99354.	30550.7	{4., 49043.}	69.2506
45	R0	{2.54943, 14.8032}	{1.43084, 16.3939}	{{1.10133, 10.3394}, {1.67375, 19.7105}}	{43.8761, 24.2385}	inf	40.	3.13333	{0., 7.}	94.5029	1.55058	newly	50.	1.85	{0., 4.}	96.3	1.89799	Reff	2.54637	1.44474	{1.1013, 1.75659}	43.2628	ar	99380.	26265.4	{1., 68335.}	73.5708
46	R0	{2.37755, 12.6461}	{1.42337, 11.4263}	{{1.0158, 8.09734}, {2.20466, 17.2398}}	{41.0287, 32.3451}	inf	8.	2.22	{0., 8.}	87.	1.5757	newly	26.	1.85	{0., 5.}	94.3462	1.90249	Reff	2.37696	1.42329	{1.01577, 2.20459}	41.0187	ar	99096.	14250.3	{1., 63489.}	85.6197
47	R0	{1.69279, 11.6636}	{2.19848, 14.9526}	{{1.86902, 14.8348}, {2.22641, 14.8348}}	{30.4036, 28.6924}	inf	138.	85.5172	{9., 205.}	61.7617	0.672756	newly	167.	52.8966	{2., 128.}	69.6676	0.827271	Reff	1.68417	2.21247	{1.86822, 2.22625}	31.5183	ar	96214.	81086.3	{75216., 85350.}	15.723
48	R0	{1.82402, 10.0038}	{1.43602, 16.3948}	{{1.11164, 8.84894}, {1.67375, 19.7105}}	{22.5496, 68.824}	inf	25.	3.59615	{0., 7.}	89.1026	1.39648	newly	39.	1.86538	{0., 5.}	95.217	1.81788	Reff	1.82252	1.43806	{1.11155, 1.75659}	22.3313	ar	97076.	30500.9	{1., 68346.}	68.5804
49	R0	{1.6424, 11.1667}	{2.90125, 15.001}	{{2.44675, 11.6528}, {2.99911, 15.963}}	{76.6467, 35.8045}	inf	267.	780.69	{80., 1544.}	139.591	0.44127	newly	380.	574.732	{67., 1154.}	100.474	0.411805	Reff	1.62686	2.85199	{2.43782, 2.99188}	75.3059	ar	95546.	91504.2	{88505., 94094.}	4.23019
50	R0	{2.05638, 14.3048}	{2.35798, 15.6935}	{{1.34361, 11.6151}, {2.86501, 18.4249}}	{28.9698, 21.9395}	inf	167.	46.4	{2., 127.}	79.0045	1.06655	newly	176.	30.1	{0., 72.}	82.8977	1.17891	Reff	2.0461	2.50936	{1.51222, 2.86467}	31.7552	ar	98194.	77030.7	{10., 93201.}	21.5525
